# Acid-Base Status Disturbances in Patients on Chronic Hemodialysis at High Altitudes

**DOI:** 10.1155/2018/2872381

**Published:** 2018-11-18

**Authors:** Javier Enrique Cely, Oscar G. Rocha, María J. Vargas, Rafael M. Sanabria, Leyder Corzo, Roberto E. D'Achiardi, Eduardo A. Zúñiga

**Affiliations:** ^1^Renal Therapy Service (RTS), Bogotá 110221, Colombia; ^2^Hospital Universitario Nacional de Colombia, 111321 Bogotá, Colombia; ^3^Assistant Professor, Internal Medicine Department, Universidad Nacional de Colombia, Colombia

## Abstract

**Background:**

Acid-base disorders have been previously described in patients with chronic hemodialysis, with metabolic acidosis being the most important of them; however, little is known about the potential changes in acid-base status of patients on dialysis living at high altitudes.

**Methods:**

Cross-sectional study including 93 patients receiving chronic hemodialysis on alternate days and living in Bogotá, Colombia, at an elevation of 2,640 meters (8,661 feet) over sea level (m.o.s.l.). Measurements of pH, PaCO_2_, HCO_3_, PO_2_, and base excess were made on blood samples taken from the arteriovenous fistula (AVF) during the pre- and postdialysis periods in the midweek hemodialysis session. Normal values for the altitude of Bogotá were taken into consideration for the interpretation of the arterial blood gases.

**Results:**

43% (n= 40) of patients showed predialysis normal acid-base status. The most common acid-base disorder in predialysis period was metabolic alkalosis with chronic hydrogen ion deficiency in 19,3% (n=18). Only 9,7% (n=9) had predialysis metabolic acidosis. When comparing pre- and postdialysis blood gas analysis, higher postdialysis levels of pH (7,41 versus 7,50, p<0,01), bicarbonate (21,7mmol/L versus 25,4mmol/L, p<0,01), and base excess (-2,8 versus 2,4, p<0,01) were reported, with lower levels of partial pressure of carbon dioxide (34,9 mmHg versus 32,5 mmHg, p<0,01).

**Conclusion:**

At an elevation of 2,640 m.o.s.l., a large percentage of patients are in normal acid-base status prior to the dialysis session (“predialysis period”). Metabolic alkalosis is more common than metabolic acidosis in the predialysis period when compared to previous studies. Paradoxically, despite postdialysis metabolic alkalosis, PaCO_2_ levels are lower than those found in the predialysis period.

## 1. Introduction

Advanced chronic kidney disease is associated with several disorders of the acid-base balance, particularly, chronic metabolic acidosis. Additionally, three decades ago, changes in ventilatory and metabolic parameters were described during hemodialysis, such as increased minute ventilation, increased CO2 excretion, increased oxygen consumption, and increased pH and HCO3 levels in the postdialysis period [1, 2].

Nowadays, most of the research conducted on this topic have focused on showing the importance of HCO_3_ or pH measurements and establish therapeutic goals for patients on dialysis [3–5]. Although these publications describe a complex association between mortality and predialysis HCO_3_ or pH levels, a more comprehensive assessment of the acid-base status based on a joint interpretation of pH, PaCO_2_, and HCO_3_ values have been recently introduced; a comprehensive approach to these characteristics was adopted by Marano M. et al., who documented the occurrence of respiratory acid-base disorders in up to 41% of cases in addition to metabolic acidosis, which cannot be identified when total CO_2_ or HCO_3_ are examined separately. Unfortunately, this research does not include data from patients living at high altitude [6].

When assessing the acid-base status in patients on dialysis, it must be taken into account that the physiological response to respiratory disorders is abnormal, especially in anuric patients; therefore, serum bicarbonate levels depend on the HCO3 provided by dialysate bath; this implies a need for adjusting the HCO3 concentration in the dialysate bath based on associated respiratory disorders in order to avoid detrimental effects caused by the therapy (e.g., cardiac arrhythmias in patients with respiratory alkalosis who receive high loads of bicarbonate with the dialysate bath in order to achieve a goal of serum HCO3 greater than 22mmol/L). However, despite knowing the effects of altitude on ventilatory physiology, previous studies did not take this factor into account; thus, pH and bicarbonate targets for dialysis patients could be altitude-dependent [6, 7].

Previously, Bejarano et al. developed a study in a population of 8 patients with chronic hemodialysis residents in Bogotá (a city with an elevation of 2,640 m.o.s.l.). The reported results showed a high prevalence of metabolic acidosis and higher partial pressures of oxygen when compared with a healthy population; nevertheless, the conditions of the dialytic treatment were different from those of today, since the standard hemodialysis solution contained 36.6 mmol/L of acetate and high-flux; high-efficiency filters were not available [8].

Therefore, the purpose of this study is to describe acid-base disorders in patients on chronic hemodialysis living in Bogotá, which is a city located at 2,640 meters over sea level.

## 2. Materials and Methods

### 2.1. Study Design and Patients

A cross-sectional study was designed by RTS, Instituto Nacional del Riñón in Bogotá. A convenience sampling included 93 patients >18 years old from the chronic hemodialysis program (receiving therapy on alternate days), with a functional arteriovenous fistula or polytetrafluorethylene (PTFE) graft, and who had been on therapy for at least 3 months. Patients were excluded if they were hospitalized or had major surgery, severe acute disease, and/or active infection in the month preceding their enrollment in the study. Likewise, patients with active neoplasia, chronic inflammation syndrome associated with kidney transplant, dysfunctional arteriovenous fistula (defined by a blood pump flow rate (QB) <250 mL/min, or recirculation percentage >10%) and users of transient or tunneled catheters intended for use in hemodialysis were also excluded, as well as those who did not provide their consent.

Hemodialysis therapy was performed three times a week with a Phoenix® machine from Gambro, using high-flux, high-efficiency dialyzers (Revaclear 300 and 400 or Polyflux 140H) and standard dialysis solution containing: bicarbonate 34 mmol/L, potassium 2 mEq/L, chloride 109,5 mEq/L, calcium 1,75 mEq/L, magnesium 0,5 mEq/L, and acetic acid 3,0 mmol/L. Sodium conductivity was adjusted according to medical prescription, based on the clinical needs of the patient, ranging from 13,5 to 13,8 mS/cm^2^. Bicarbonate conductivity settings were not adjusted in the 3 months prior to enrolling in the study and during the study.

### 2.2. Method for the Collection and Processing of Blood Samples

Blood samples were collected from the arterial side of the arteriovenous fistula using 1 mL heparinized syringes in the pre- and postdialysis periods and in the midweek session; blood processing was done immediately after the sampling by a standard method using a RAPIDLab® 348 EX system from Siemens. Simultaneously, blood samples were taken to perform monthly control tests required by the renal clinic, which became analytical variables to be discussed in our study.

### 2.3. Variables

Clinically relevant variables included in the study were age, sex, time on dialysis with chronic kidney disease stage 5 and etiology, dry weight, pre- and postdialysis systolic and diastolic blood pressure, dialyzer, monocompartment Kt/V (Single Pool), malnutrition inflammation score (MIS), normalized protein catabolic rate (nPCR), and daily protein, carbohydrate, and fat intake. The use of chelation therapy with sevelamer, calcium carbonate, and aluminum hydroxide was documented in the medication history. Laboratory data taken into account were intact hormone parathyroid (PTHi) levels, calcium, phosphorus, potassium, albumin, and complete blood count. The sample for blood gas analysis was tested for pH, partial pressure of carbon dioxide (PaCO_2_), partial pressure of oxygen (PaO_2_), bicarbonate (HCO_3_), oxygen saturation (SO_2_), and base excess (BE).

The interpretation of arterial blood gases was based on normal values for Bogotá, a city located at 2,640 m.o.s.l. according to the study by Maldonado et al. [9] and the identification of primary acid-base disorders was standardized in accordance with the eight possibilities proposed on the Siggaard-Andersen nomogram in 1963, which were further adjusted for altitude by P.E Paulev in 2005 [7, 10]. For practical purposes, all acid-base disturbances are referred to as “acidosis or alkalosis”, without making any distinction with regard to the terms “acidemia and alkalemia”.

Details for the interpretation of arterial blood gases are out of the scope of this manuscript; it is recommended to refer to the article by Lasso JI, “*Interpretación de los gases arteriales en Bogotá (2.640 msnm.) basada en el nomograma de Siggaard-Andersen. Una propuesta para facilitar y unificar la lectura*” [11].

### 2.4. Statistical Methods

Blood gas data and clinical variables from Versia (electronic medical record) were collected in a database in Excel 2016. The statistical analysis was performed using STATA14® (StataCorp. 2015.* Stata Statistical Software: Release 14*. College Station, TX: StataCorp LP.). A descriptive analysis of sociodemographic and clinical variables of the population was made. Absolute and relative frequencies were measured for categorical variables. Measures of central tendency and dispersion were calculated for continuous variables based on their distribution. Student t-test and Wilcoxon signed-rank test were used for comparative analysis of the arterial gasometric and laboratory variables obtained in the pre- and postdialysis period, according to the distribution of variables. P value <0.05 was considered statistically significant.

### 2.5. Ethical Considerations

This paper was submitted and approved by the RTS Research Ethics Committee.

## 3. Results

A total of ninety-three patients were included. 68,8% (n=64) were male. The median age was 60,9 years (IQR 50,8 – 71,7). The mean time on dialysis was 48.5 months (IQR 34,6–88,4). The most frequent cause of chronic kidney disease was diabetes mellitus in 38,7% (See Table 1).

Average pre- and postdialysis pH values were 7,41 (IQR 7.39–7.43) and 7,51 (IQR 7,49–7,54), respectively; statistically significant differences were found in pH, PaCO_2_, HCO_3_, BE, and SO_2_ between pre- and postdialysis periods (p<0,01). No differences were found between pre- and postdialysis PaO_2_ levels (p=0,096) (see Table 2).

When interpreting the values of predialysis arterial blood gases, normal acid-base status was the most common with 43% (n=40), followed by metabolic alkalosis with chronic hydrogen ion deficiency in 19,6% (n=18), with an average nPCR of 1,03 (±0,19) and MIS of 5 (IQR 4 – 6). All patients experienced postdialysis metabolic alkalosis (see Table 3).

## 4. Discussion

This study reports the acid-base status of patients on hemodialysis at an altitude of 2640 meters above sea level. It should be highlighted that the interpretation of acid-base status at altitude and high altitude has to consider that residents are in a normal and balanced status for each altitude and this can bring about misinterpretation of the actual acid-base status, if sea level reference values are used [12]. Therefore, one of the strengths of this work is the interpretation of arterial blood gases using reference values adjusted to Bogotá's altitude.

The results of this study show that almost half of the patients are in normal acid-base status and the most common disorder is predialysis chronic metabolic alkalosis. This differs from previous publications where chronic metabolic acidosis is most frequently due to the interdialytic accumulation of volatile acids [2, 6, 8]. The findings of this study could be explained by the HCO_3_ accumulation provided by the dialysate bath with a poor renal compensatory response in advanced chronic kidney disease. So far, we ignore whether this susceptibility to alkalosis and high altitude has any impact on the dialysis outcomes and whether there is any benefit in tailoring the bicarbonate bath concentration by the altitude. This is important, since some recent literature reported improved survival for patients on chronic dialysis living above 1,800 m.o.s.l., which has not been clearly explained [13].

On the other hand, we rule out that metabolic alkalosis is secondary to chronic inflammation and poor protein intake since no major differences were found in nPCR values (close or equal to 1g/Kg) between the groups with acid-base disturbances, and the great majority showed malnutrition inflammation scores of less than 8 points. Also, the bulk of our patients have proper levels of [CO_2_ total] (extrapolated to HCO_3_) according to the targets recently proposed by Gennari F.J of 18 mEq/L to 26 mEq/L; these values are less stringent compared to the K/DOQI guidelines (>22mEq/L) that have not changed in the last 18 years [14, 15].

Furthermore, despite postdialysis metabolic alkalosis, PaCO_2_ levels paradoxically decreased as compared to predialysis values. An explanation for this might be that dialysis therapy with high-flux, high-efficiency filters, and bicarbonate bath dialysate produce substantial blood amounts of CO_2_ that stimulate the respiratory center, increasing minute ventilation and resulting in lower levels of PaCO_2_ at the end of the therapy [1].

This study has some limitations which have to be pointed out. First, the variables residual renal function and use of diuretics—which may be modifiers of the acid-base status—were not available. Secondly, there are not data available on cardiopulmonary comorbidity, which is critical for a better characterization of the ventilatory disorders found in our population. Thirdly, it should also be borne in mind that in our study HCO_3_ levels were calculated from pH and PaCO_2_ values by the blood gas analyzer machine using the Van Slyke equation; therefore, actual HCO_3_ values, i.e., taken by direct measurement, could differ from the estimated HCO_3_ value and they also may not be correlated to the pH and PaCO_2_ levels.

## 5. Conclusion

A substantial share of our patients on chronic hemodialysis has a normal acid-base status. At hight altitude (2,640 m.o.s.l), the most common acid-base disorder before the hemodialysis treatment was metabolic alkalosis. Paradoxically, despite postdialysis metabolic alkalosis, PaCO_2_ levels are lower than those found in the predialysis period.

## Figures and Tables

**Table 1:** Patient characteristics.

**Variable**	**Number of patients**
**n(**%**), median (IQR∗) or mean (±SD†)**	**n=93**
Age (years)	60,9 (50,8 – 71,1)

Male gender	64 (68,8)

Time on dialysis (months)	48,5 (34,6 – 88,4)

Dry weight (Kg)	63 (54 – 73)

Interdialytic weight gain (Kg)	1,5 (1 – 2,3)

**Etiology of CKD**	
Diabetes mellitus	36 (38,7)
Hypertension	31 (33,3)
Primary glomerulopathy and autoimmune disease	10 (10,7)
Obstructive	3 (3,23)
Polycystic kidney disease	6 (6,46)
Other causes	7(7,53)

**Dialysis related parameters **	
Pre-dialysis systolic blood pressure (mmHg)	132 (± 21,3)
Pre-dialysis diastolic blood pressure (mmHg)	74,8 (± 16,6)
Post-dialysis systolic blood pressure (mmHg)	133 (± 20,2)
Post-dialysis diastolic blood pressure (mmHg)	73 (± 12,3)
Therapy time (minutes)	244 (± 14,1)
Dialyzer	
Revaclear 300	42 (45,2)
Revaclear 400	46 (49,5)
Polyflux 140H	5 (5,38)
Type of extracorporeal system anticoagulation	
Unfractionated heparin	86 (92,5)
Low molecular weight heparin	4 (4,3)
No anticoagulation	3 (3,23)
Single pool Kt/V	1.57 (1.43 – 1,8)
nPCR	1,04 (± 0.2)
Malnutrition-Inflammation Score (MIS)	5 (4 – 7)
Protein intake (grams/day)	68 (58 – 75)
Lipid intake (grams/day)	42,6 (± 12,5)
Carbohydrate intake (grams/day)	197 (± 44,2)

**Laboratory parameters**	
Serum albumin (gr/dL)	4,21(± 0,31)
PTHi ‡ (ng/mL)	388 (242 – 573)
Calcium (mg/dL)	8,75 (±0,62)
Phosphate (mg/dL)	4,63 (±1,19)
Potassium (mEq/L)	5 (± 0,63)
Hemoglobin (g/dL)	12,3 (± 1,64)

**Phosphate binding therapy **	
Calcium carbonate	66 (71)
Aluminum Hydroxide	43 (46,2)
Sevelamer Chlorhydrate	4 (4,3)

*∗*Interquartile Range (IQR).

†Standard deviation.

‡Intact parathyroid hormone (PTHi).

**Table 2:** Arterial blood gases values in the pre- and postdialytic period.

**Parameter**	**Predialysis**	**Postdialysis**	**p-value**
**pH**	7,41 (7,39 – 7,44)	7,51 (7,49 – 7,54)	<0,01

**PaCO2**	35,2 (31,9 – 37,8)	33,5 (30,4 – 34,7)	<0,01

**HCO3**	21,7 (20,4 – 23,4)	25,4 (24,7 – 26,6)	<0,01

**PaO2**	65,2 (60,8 – 74,4)	68,4 (63,4 – 76,6)	0,096

**BE**	-2,8 (-4,4 – -0,6)	2,4 (1,2 – 3,8)	<0,01

**SO2**	93,3 (91,5 – 95,3)	95,4 (94,6 – 96,4)	<0,01

**Table 3:** Arterial blood gas interpretation with MIS^*∗*^ score and nPCR^†^ values.

**Acid base disorder**	**Number of patients n(**%**)**	**MIS**	**nPCR**
		**M (IQR)**	$\bar{x}$ ** (DE)**
**Pre-dialytic sample**			
Acid base equilibrium	40 (43)	5 (3 – 7)	1,06 (±0,18)
Metabolic alkalosis with chronic hydrogen ion deficit	18 (19,6)	5 (4 – 6)	1,03 (±0,19)
Chronic hypercapnic respiratory acidosis	9 (9,7)	5 (4 – 9)	0,97 (±0,25)
Acute hypercapnic respiratory acidosis	8 (8,6)	5,5 (4,5 – 11,5)	1,15 (±0,26)
Chronic metabolic acidosis with hydrogen ion excess	7 (7,53)	7 (2 – 9)	0,96 (±0,21)
Acute hypocapnic metabolic alkalosis	7 (7,53)	5 (5 – 10)	0,98 (±0,18)
Metabolic acidosis with acute hydrogen ion excess	2 (2,15)	4 (3 – 5)	1,12 (±0,18)
Chronic hypocapnic metabolic alkalosis	2 (2,15)	4,5 (3 – 6)	1,04 (±0,24)

**Post-dialytic sample**			
Metabolic alkalosis	62 (66,7)	5 (4 – 7)	1,04 (±0,19)
Metabolic and respiratory alkalosis	22 (26,7)	5,5 (3 – 8)	1,03 (±0,22)
Metabolic alkalosis and respiratory acidosis	9 (9,7)	9 (4 – 11)	1,05 (±0,23)

^*∗*^Malnutrition-Inflammation Score reported in median and Interquartile Ranges.

^†^Normalized protein catabolic rate reported as mean and standard deviation.

## Data Availability

The data used to support the findings of this study are available from the corresponding author upon request.
